# Reconstruction of the temporal signaling network in *Salmonella*-infected human cells

**DOI:** 10.3389/fmicb.2015.00730

**Published:** 2015-07-20

**Authors:** Gungor Budak, Oyku Eren Ozsoy, Yesim Aydin Son, Tolga Can, Nurcan Tuncbag

**Affiliations:** ^1^Department of Health Informatics, Graduate School of Informatics, Middle East Technical UniversityAnkara, Turkey; ^2^Department of Computer Engineering, College of Engineering, Middle East Technical UniversityAnkara, Turkey

**Keywords:** phosphoproteomic, network reconstruction, *Salmonella* infection, temporal data integration, pathway analysis

## Abstract

*Salmonella enterica* is a bacterial pathogen that usually infects its host through food sources. Translocation of the pathogen proteins into the host cells leads to changes in the signaling mechanism either by activating or inhibiting the host proteins. Given that the bacterial infection modifies the response network of the host, a more coherent view of the underlying biological processes and the signaling networks can be obtained by using a network modeling approach based on the reverse engineering principles. In this work, we have used a published temporal phosphoproteomic dataset of *Salmonella*-infected human cells and reconstructed the temporal signaling network of the human host by integrating the interactome and the phosphoproteomic dataset. We have combined two well-established network modeling frameworks, the Prize-collecting Steiner Forest (PCSF) approach and the Integer Linear Programming (ILP) based edge inference approach. The resulting network conserves the information on temporality, direction of interactions, while revealing hidden entities in the signaling, such as the SNARE binding, mTOR signaling, immune response, cytoskeleton organization, and apoptosis pathways. Targets of the *Salmonella* effectors in the host cells such as CDC42, RHOA, 14-3-3δ, Syntaxin family, Oxysterol-binding proteins were included in the reconstructed signaling network although they were not present in the initial phosphoproteomic data. We believe that integrated approaches, such as the one presented here, have a high potential for the identification of clinical targets in infectious diseases, especially in the *Salmonella* infections.

## Introduction

*Salmonella enterica* is a gastrointestinal pathogen that infects the human cells by translocation of its effector proteins with a vacuole called the *Salmonella* containing vacuole (SCV). The SCV compartment allows the pathogen to replicate and proliferate within the host cells. The secretion system transfers the pathogen proteins, which are called the effectors, directly into the cytosol of the host cells (reviewed in Dandekar et al., 2012). Translocation is achieved by type III secretion systems (T3SSs) where T3SS-1 is responsible for the regulation and replication of the SCV. These effectors have interactions with the host proteins and can change cell functions such as apoptosis, post-translational modifications, and intracellular signaling. *Salmonella* can adapt to a broad range of environmental conditions and process many different metabolites (Dandekar et al., 2012). Although many efforts have been invested to understand the adaptation mechanism of *Salmonella*, functions of its effector proteins, the affected metabolic regulatory pathways, details of the host-pathogen communication and the changes in the host signaling pathways are still unknown. A systems level modeling approach has been performed on the effectors of *Salmonella* to understand the adaptation process and 14 regulators have been identified to play a critical role in the regulation of the genes responsible for *Salmonella* infection (Yoon et al., 2009). Also, the *Salmonella*'s metabolic network during its replication has been modeled using flux balance analysis, which has led to the identification of a set of metabolic pathways crucial during the intracellular replication (Raghunathan et al., 2009). The invasion of the pathogen is mainly transduced by the protein kinase signaling cascades in the host cell. *Salmonella* infection promotes apoptosis and adapts to the host cell's ubiquitination process (Steele-Mortimer, 2011). Regarding the regulome of *Salmonella*, the context likelihood of relatedness (CLR) approach has been used to infer the transcriptional regulatory connections by using mutual information in gene expression data and several regulatory networks have been identified (Taylor et al., 2009).

Understanding the communication and the signaling between *Salmonella* and its host in detail is crucial to improve the available treatment strategies for the *Salmonella* infection. The recently released interactome of the *Salmonella* effectors and human proteins, which has been curated from the literature, again revealed the enrichment of the MAPK signaling and the apoptotic pathways for the studied protein set (Schleker et al., 2012). The advances in high-throughput omic technologies also allow the systems-level identification of signaling components within the host cell. The analysis of mRNA expression of ~4300 genes after a *Salmonella* infection in the human epithelial cells showed that NF-κB is a key transcription factor in the regulation of a wide range of genes (Eckmann et al., 2000). Also several cytokines, transcription factors and kinases are shown to be over-expressed in the same study. In a temporal gene expression analysis, where the Bayesian network analysis is used, the immune response, Wnt, PI3K, mTOR, TGF-β, and many other signaling pathways were found to be altered during the *Salmonella* infection. The host signal response was shown to be activated during the earlier time points rather than later (Lawhon et al., 2011). In another study, different gene expression datasets were integrated with protein–protein interactions and compared to each other to find out the specific subnetworks altered by *Salmonella* infection in the host (Dhal et al., 2014). In a global temporal phosphoproteomic analysis of *Salmonella*-infected human cells, 9500 phosphorylation events were quantified during the first 20 min of the infection and regulated host pathways were identified. Clustering analysis showed that the effector SopB was mainly responsible for the alterations of the phosphorylation events in the host cell (Rogers et al., 2011). Although omic technologies provide large amount of high dimensional data, the complete map of the signaling pathways cannot be retrieved by the direct connections of omic hits, as there are many intermediates which are not represented in the experimental data. Signaling networks can also be modeled by optimization based approaches (Dittrich et al., 2008; Huang and Fraenkel, 2009; Yeger-Lotem et al., 2009; Gosline et al., 2012; Huang et al., 2012; Tuncbag et al., 2013) where omic hits are defined as constraints. Previously, various network modeling approaches have been applied for the integration of the multiple omic sets of diseases and the disease networks of various cancer types have been successfully reconstructed (Kim et al., 2011; Huang et al., 2012). Regulatory networks can be reconstructed by various approaches, utilizing the gene expression data, including Boolean networks, Bayesian networks, and methods based on information theory and differential equations (reviewed in detail in De Jong, 2002; Hecker et al., 2009; Linde et al., 2015). Analysis of the perturbation data have also been proposed for the reconstruction networks (Markowetz et al., 2007; Frohlich et al., 2009; Bender et al., 2010; Aijo et al., 2013; Kiani and Kaderali, 2014). For example Nested Effects Models (NEMs) (Markowetz et al., 2007) use a set of knocked-down genes and their indirect effect on a larger set of genes to reconstruct the network. Methods that utilize observations of perturbed networks at a steady state or at several time points include (Dynamic) Deterministic Effects Propagation Networks [(D)DEPNs] (Frohlich et al., 2009; Bender et al., 2010), Sorad (Aijo et al., 2013), and Dynamic Probabilistic Boolean Threshold Networks (DPTBNs) (Kiani and Kaderali, 2014). However, these network reconstruction methods are computationally expensive and do not scale well for the reconstruction of large networks. Recently, Linear Programming (LP) based approaches have also been used to solve the network reconstruction problem (Eren Ozsoy and Can, 2013; Knapp and Kaderali, 2013; Matos et al., 2015). LP-based methods model the reconstruction problem as an optimization problem and are able to construct networks from both perturbation and time-series assays. However, based on the optimization function and the linear constraints, LP-based methods may be computationally expensive, as well. For example, a very recent method, lpNet (Matos et al., 2015), requires 3 days to reconstruct a 20 node network in the *in silico* dataset of the HPN-DREAM breast cancer network inference challenge. The DREAM (Dialogue on Reverse-Engineering Assessment and Methods) challenge aims to setup a joint effort between computational and experimental biologists toward revealing the cellular networks from multiple high-throughput data (Stolovitzky et al., 2007). An LP variant, the Integer Linear Programming (ILP) approach, by Melas et al. uses several optimization steps to find and remove the inconsistencies between measurements and the input network topology (Melas et al., 2013). Additionally, ILP is a known NP-hard problem, and due to the large number of variables, this method may not find solutions in a reasonable time, as it requires 64.000 s for a 14 node network. We have previously proposed a divide and conquer based ILP solution for the perturbation data analysis, which scales well for larger networks by merging the solutions of the smaller sub-networks (Eren Ozsoy and Can, 2013). The main difference of our proposed ILP approach was the definition of the optimization function as the minimization of the discrepancy between a reference network and the inferred network. Recently we have extended our ILP approach to work on time series data (Eren Ozsoy et al., 2015) and here we have directly apply that method for the construction of the temporal signaling network in *Salmonella*-infected human cells. Although network modeling approaches are easily adaptable to identify signaling components in various disease states, to our best knowledge, these approaches have not yet been applied for the reconstruction of signaling networks in the human host cell during the *Salmonella* infection.

In this work, the temporal phosphoproteomic data of the *Salmonella*-infected human cells (Rogers et al., 2011) have been used to model the altered signaling network in the host cells. We have used a powerful combination of two different network modeling approaches to construct the signaling network of *Salmonella*-infected human cells. First, the temporal phosphoprotemic data of *Salmonella*-infected human cells are integrated with protein interaction data to construct the signaling pathway at each time point. Then, all constructed networks were merged together, and used as the input for the second part of the network modeling step, in which directions are assigned to the interactions based on the temporal data. Our approach allowed us to identify host pathways altered during *Salmonella* infection. In addition, by using network analysis techniques, we have provided a ranking of the proteins according to their importance during the infection.

## Materials and methods

### Datasets

We have used the global temporal phoshoproteomic dataset published in (Rogers et al., 2011), where four time points, 2, 5, 10, and 20 min after *Salmonella* infection in human cell, were selected. Another dimension of this dataset is the cellular compartments where the phosphorylation site is identified as membrane, cytosol, or nucleus. At each time point, if the change in the phosphorylation status of a peptide is significantly altered compared to the uninfected cells (*p* < 0.05) and the variance across biological replicates are small (variance <15%) then that peptide is selected for the next step of the analysis, so added to the dataset. Then, each peptide selected, has been mapped to their HUGO Gene Nomenclature Committee (HGNC) identifiers using the Database for Annotation, Visualization and Integrated Discovery (DAVID) web server (Huang et al., 2009). If multiple peptides map to a single protein, then the peptide with the maximum value of fold change for phosphorylation level is included for further analysis.

Besides the global phoshoproteomic data, the human protein interactome is used for the data integration and modeling. The interaction data from iRefWeb has been downloaded which has 113,248 confident weighted interactions between 15,684 proteins (Turner et al., 2010). Also, a *Salmonella* effector to human host protein interactome, which consists of 40 effectors and 50 host proteins connected with 62 interactions, has been retrieved (Schleker et al., 2012).

### Network modeling

**Figure 1:**
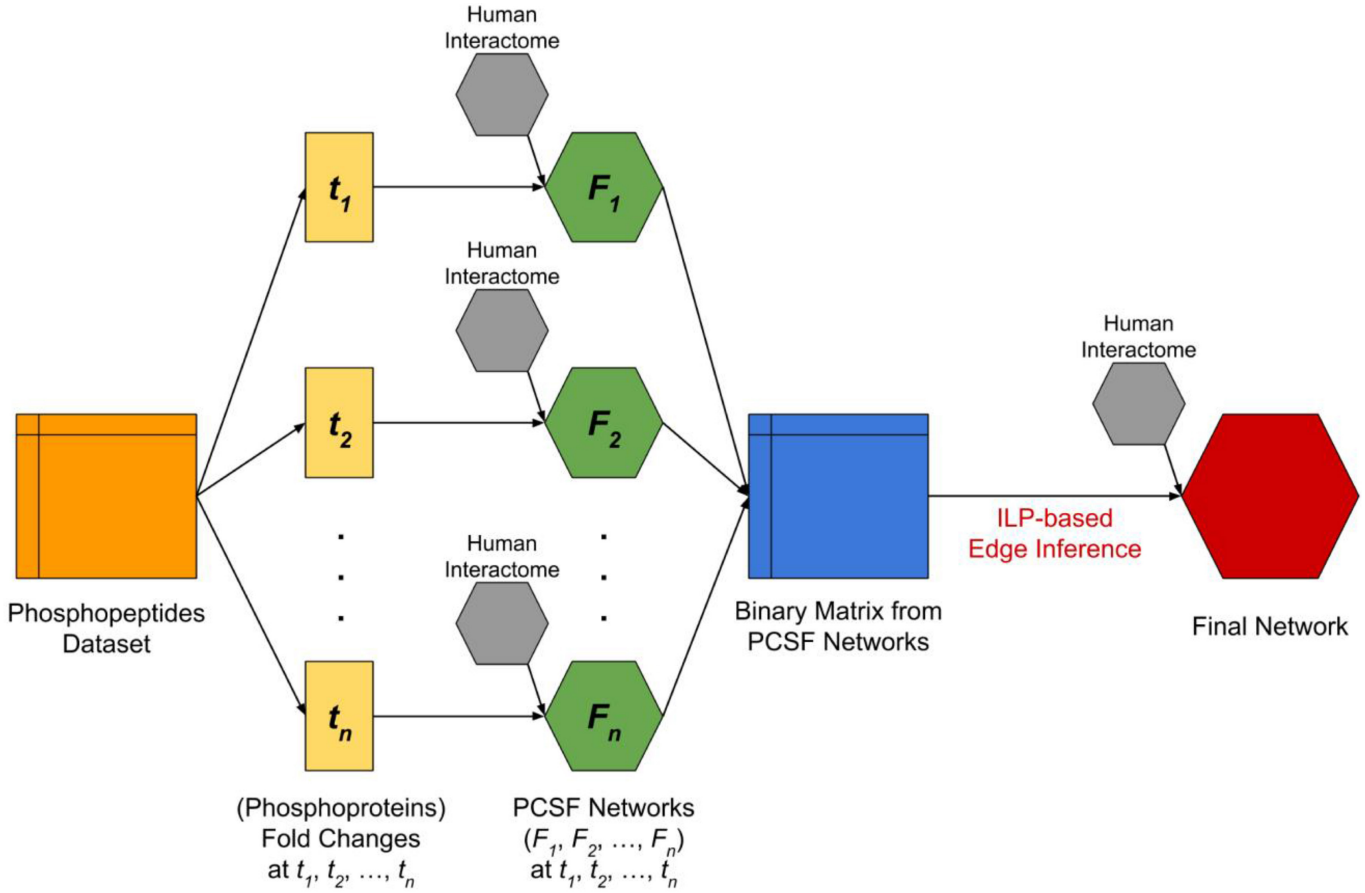
**The flowchart of complete analysis**. The dataset which includes temporal fold changes of phosphopeptides at four different time points (*t*_*1*_ = 2 min, *t*_*2*_ = 5 min, *t*_*3*_ = 10 min, *t*_*4*_ = 20 min) and at three different locations (nucleus, cytoplasm, and membrane) was split and converted into temporal fold changes datasets of the corresponding phosphoproteins by taking the maximum fold change among phosphopeptides that were observed at different locations and mapped to the same phosphoprotein. Next, we applied PCSF approach for each fold changes dataset by integrating human interactome in order to discover hidden intermediate proteins. The resulting networks (*F*_*1*_, *F*_*2*_, *F*_*3*_, *F*_*4*_) are then used to form a binary matrix where the rows are time points and columns are phosphoproteins. Each corresponding cell of the binary matrix represents a significant change (*p* < 0.05 and variance <15%) in the phosphoprotein at the time point. Finally, we applied an ILP-based edge inference approach by integrating human interactome in order to validate and determine edges and edge directions.

The network modeling procedure is composed of two stages; (i) network construction using the Prize-collecting Steiner Forest (PCSF) approach, and (ii) network reconstruction using the ILP based edge inference approach. These two approaches complement each other as the PCSF approach reveals the hidden components in signaling by finding the high confidence regions in the interactome, and the ILP-based edge inference approach reconstructs interactions and their directionality by using temporal data as constraints. In Figure 1, the flowchart of our integrated approach is given.

#### Prize-collecting steiner forest approach

PCSF is based on finding the high-confidence regions within a protein interactome, which is used to recover the phosphoproteomic hits (i.e., the terminal nodes) and hidden proteins from the global temporal phoshoproteomic data (i.e., the Steiner nodes) in this study (Tuncbag et al., 2013). In the optimization stage, two objectives are important; avoiding the low-confidence protein-protein interactions in the final network and including as many phosphoproteomic hits as possible. Each protein identified with a significantly changed phosphorylation status at a time point is assigned a weight equal to the absolute value of the log fold change in phosphorylation, which is called the “prize.” The cost of an interaction is calculated from its confidence score where higher cost implies lower confidence. The algorithm has to assign a cost for each interaction included in the network, and pay a penalty for excluding a phosphoproteomic hit equal to its prize. For a given, directed or undirected network G(V, E, *c*(*e*), *p*(*v*)) with a node set V and edge set E, *p*(*v*) ≥ 0 assigns a prize to each node *v* ∈ V and *c*(*e*) > *0* assigns a cost to each edge *e* ∈ E. The aim is to find a forest F(V_*F*_, E_*F*_) that minimizes the objective function:

(1) $$f\left(t\right)=\sum_{v\;\notin \;F}\left(\beta \cdot p(v)\right)+\sum_{e\;\in \;F}\left(c(e)\right)+\omega \cdot \kappa$$

where κ is the number of trees in the forest and β is the scaling factor. Another parameter that is used at the optimization stage is the depth value (D) which represents the maximum allowed number of edges from the root node to any terminal node. To convert the PCSF problem into a Prize-collecting Steiner Tree (PCST) problem we have introduced an extra root node *v*_0_ into the network connected to each terminal node *t* ∈ T by an edge (*t*, *v*_0_) with cost ω where **T** ⊂ **V**. This optimization problem has been solved with a message passing algorithm, the msgsteiner tool (Bailly-Bechet et al., 2010). The forest F is defined as a disjoint collection of trees with all edges pointing to the roots. In this work, depth is set to 10, ω is in the interval [1, 10] and β is in the interval [1, 10]. Optimum forests obtained by each parameter combination are merged together in order to consider the suboptimal solutions. Finally, a PCSF is constructed for each time point.

#### Integer linear programming (ILP) based edge inference approach

For constructing signaling and regulatory networks using time series expression data, we have used an extended version of our previous ILP model that can handle both the time series and steady state perturbation data (Eren Ozsoy and Can, 2013). The extended ILP-based edge inference approach proposed in Eren Ozsoy et al. (2015) is highly scalable when time series data is available; therefore, in this paper, we directly apply that method for the construction of the whole *Salmonella* infection signaling network using a single integer linear program. The details of the ILP model are given below.

##### The integer linear programming model

Assuming that a reference signaling network is given as a directed graph G(V, E), where V represents the node set (i.e., proteins) and E represents the edge set (i.e., pairwise interactions), with several source nodes *s*_*i*_, and sink nodes *t*_*j*_, a reference regulatory network can be curated from literature or obtained from a public database. The steady state knock-down version of this problem has been shown to be NP-complete (Hashemikhabir et al., 2012). When the same problem is formulated as a linear optimization problem, the solution of this optimization problem provides a network, satisfying the experimental observations with minimum number of changes (insertion or deletion) of the edges on the reference network. The raw time-series expression data is assumed to be processed, and the binary activity data is available for the proteins in the network. We have used the cutoffs (*p* < 0.05 and variance <15) as described in the Datasets. Steiner nodes are also assumed to be active based on their presence in the reconstructed PCSF networks.

As the objective function of the model is to minimize the edit operations, i.e., insertions/deletions of edges, on the reference network, the proposed model also works when there is no reference network available. For such cases, the smallest network satisfying the expression data is sought. Let *x*_*ij*_ be the binary variable representing the presence of the edge from node *i* to node *j* in the reference network. If the edge is present, then the value of *x*_*ij*_ is 1, otherwise it is 0. Correspondingly, *w*_*ij*_ represents the presence of the edge from node *i* to node *j* in the network to be reconstructed from observations. For a graph G(V, E) with *n* nodes, the objective function is given in Equation (2), which basically quantifies the difference between the reference network and the reconstructed network.

In the solution phase, the matrix of state variables is used for the construction of the linear constraints. A protein is assumed to be activated once the corresponding state variable becomes 1. For the model, it does not matter what value the state variable is assigned thereafter. For the construction of the constraints, the kinematics of the system is taken into consideration. A protein is assumed to be activated by any protein, which is already activated at any previous time point and also a protein is able to activate any protein at any of the following time points. The constraints are based on the following assumptions: (1) sources are the proteins which are activated at the first time point and sinks are the proteins which are activated at the last time point, (2) each source node and sink node has to be connected to the network, (3) at each time point, the proteins may only be activated by the upstream proteins, which are active in preceding time points, (4) no direct edges from sources to sinks are allowed, (5) no edges between sources or sinks are allowed, and (6) no self-edges are allowed. Note that these assumptions do not allow an upstream edge. However, there may be such edges in the reference network which are to be removed in the reconstructed network. Based on these assumptions, the following graphical constraints are derived.

1. There should be at least one edge going out of each source protein to the proteins activated at the second time point.
2. There should be at least one edge going into each sink proteins from the proteins activated at the last time point.
3. There should be at least one edge going into an intermediate node from the upstream nodes activated at a previous time point.
4. There should be at least one edge going out of an intermediate node to one of the downstream nodes, including the sink nodes.

Note that these constraints are derived only from the time series expression data. It is also possible to add additional constraints, if any perturbation experiment is available for the network. Let the set V_*i*_ be the set of proteins active at time point *i*. Let V_*s*_ be the set of source nodes and V_*t*_ be the set of target (i.e., sink) nodes. The node set of the reconstructed network V is the union of all source nodes, target nodes, and all the nodes active at some time point. Let V_*p*_ be the set of nodes activated just before the sink nodes. Let V_*d*_ be the set of downstream nodes that are activated after the activation of node *i*. The overall Integer Liner Program is then given as:

(2) $$Minimize\sum_{i\;=\;1}^{n}\sum_{j\;=\;1}^{n}\left|x_{ij}-w_{ij}\right|$$

Subject to:

(3) $$\sum_{j\;\in \;V_{1}}x_{ij}\geq 1\text{ for all }i\in V_{s}$$

(4) $$\sum_{i\;\in \;V_{p}}x_{ij}\geq 1\text{ for all }j\in V_{t}$$

(5) $$\sum_{j\;\in \;V_{d}}x_{ij}\geq 1\text{ for all }i\in V_{x}$$

(6) $$\sum_{i\;\in \;(V\backslash V_{s})}x_{ij}\geq 1\text{ for all }j\in V_{s\;+\;1}$$

### Assessment of the improvement

In the first step, we only use the temporal phosphoproteomic data and reconstruct the signaling network without a reference network. Then, PCSFs for each time point are merged together and a binary matrix has been created from the PCSF network in order to validate the edges and to determine edge directions the ILP based edge inference approach is used. The human protein interactome described in Datasets is assigned as the reference network for the ILP analysis. So, the PCSF and ILP based edge inference analysis are combined to provide the intermediate nodes (from the human interactome) based on the proteins identified at different time points from the experimental data, in addition to the direction information for the edges. The resulting directed network is then used for visualization and further analyses.

### Network analysis and clustering

Restricted Neighborhood Search Cluster Algorithm (RNSC) in the NeAT toolbox (Brohee et al., 2008) was used to cluster the network where the maximum number of clusters was selected to be 20 and other parameters were kept as the default values. Critical nodes in the network were ranked by calculating four attributes: the path frequency, in-degree, out-degree, the sum of in-degree and out-degree, and the betweenness centralities. A simple path is an ordered sequence of nodes in a graph such that each node occurs at most once in this sequence and each pair of consecutive nodes is connected by an edge. Given all the possible simple paths between the terminal nodes at 2 min and the terminal nodes at 20 min, the path frequency of a node *p* is defined as the ratio of the simple paths that include *p* over all paths. The network analysis has been performed by using the Python NetworkX package (Schult and Swart, 2008).

### Functional enrichment analysis

After clustering the network with RNSC algorithm, enrichment of each cluster has been assessed with DAVID web server in the following categories: biological process ontology, cellular component ontology, molecular function ontology, BBID pathways, BIOCARTA pathways, and KEGG pathways. Then, we have collected the enrichment results for each cluster and generated a matrix where rows are Gene Ontology (GO) terms and columns are corresponding *p*-values for each cluster, for all the data with *p* < 0.05, and enriched proteins >1%.

## Results

### Reconstruction of temporal signaling networks in *Salmonella*-infected human cells

Modeling signaling networks is a more challenging task when the time dimension is taken into account. In a given temporal omic data, one of the problems to be solved is how the omic hits are connected and what are the upstream and downstream regulators in the final signaling network. The ILP based edge inference approach uses the temporal information as constraints and reconstructs edges and their direction between the omic hits toward solving this problem. But ILP-based edge inference approach cannot identify the missing components in the omic hits for the representation of the whole signaling system. The PCSF approach solves this problem by searching for the most confident region of the interactome that will include most of the experimental hits and adds hidden components, called Steiner nodes. As a limitation, if the initial interactome is undirected, the PCSF approach cannot assign directions to the edges and cannot use the temporal constraints, which can be solved by the ILP-based edge inference approach. As the different aspects of the ILP-based edge inference and PCSF network modeling approaches are complementing each other, we have combined these two approaches to reconstruct the temporal signaling networks in *Salmonella*-infected human cells.

**Figure 2:**
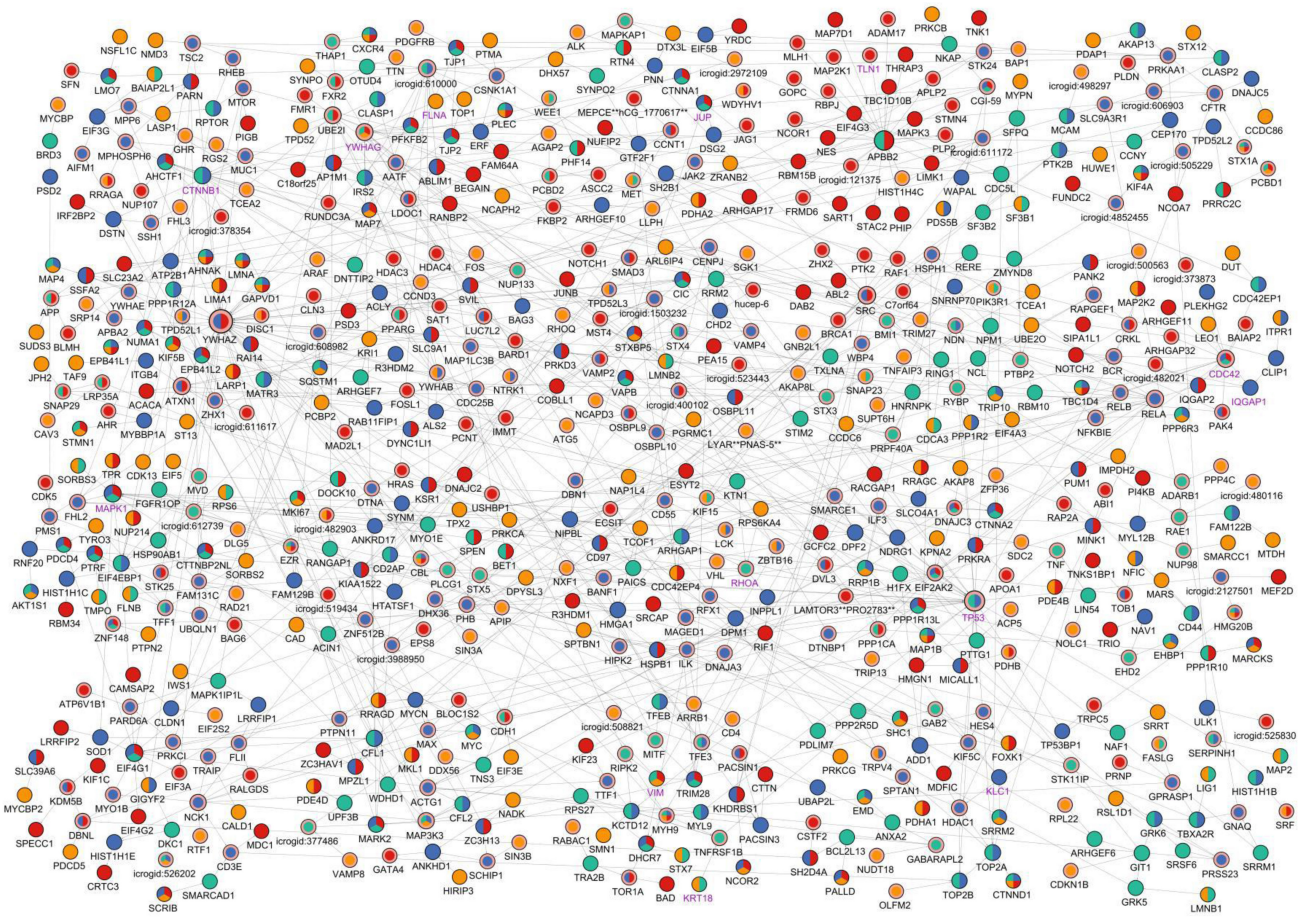
**Visual representation of the reconstructed signaling network of** ***Salmonella*****-infected host cell**. The reconstructed network is clustered (Cluster #1 is at the left-top and Cluster #20 is at the right-bottom) and the layout of the network has been arranged accordingly for visualization. Time points when any node is found to be critically changed are shown with different colors on the nodes; yellow indicates a change at 2 min in the node, green indicates change at 5 min, blue indicates change at 10 min, and red indicates change at 20 min. If a node is present at more than one time points, then its coloring is a combination of colors of the corresponding time points. Steiner nodes differ from terminal nodes by a pink border and *Salmonella*—host interactome nodes differ from host nodes with a purple node label. Node sizes linearly proportional to the sum of in degree and out degree values of the nodes.

In this work, we have used the phosphoproteomic data published in (Rogers et al., 2011). This dataset has been first divided into three parts at each time point based on the cellular compartment (membrane, cytosol, and nucleus). Additionally, the overlaps of the significantly phosphorylated proteins across different time points and different cellular compartments are compared. We have noted that the overlap between compartments within the same time points were very small, so the phosphoproteins at the same time point but in different compartments are safely merged (see Table S1). Next, for each time point, we have prepared a set of significantly phosphorylated proteins along with their fold changes during phosphorylation. To show the importance of revealing hidden components that were not present in the phosphoproteomic hit set, we first ran only the ILP-based edge inference approach on the data. The result was a disconnected network with many small sub-networks composed of three or four nodes which were not a representation of any pathway (Figure S1). This visual representation clearly showed that experimental hits alone are not enough to represent the complete network as there are missing components between these nodes. At this point, we took advantage of the PCSF approach in revealing hidden nodes and prepared a reference network to be used in the ILP-based edge inference approach. For this purpose, multiple PCSFs have been constructed for each time point and merged together to form a single network. To pipe this output into the ILP-based edge inference approach, we have prepared a network matrix where rows are proteins, columns are time points. When a node is present at a time point either as a phosphoproteomic hit or as a hidden node revealed by the PCSF approach, it is labeled as 1, otherwise it is 0. The ILP-based edge inference approach reconstructed a network based on the provided matrix as a reference network. The final network was composed of 658 nodes and 869 edges. As shown in Figure 2, the resulting network keeps the temporal information and also reveals hidden proteins and directions of the interactions. An interactive visualization and related source information is available at http://mistral.ii.metu.edu.tr/salmonella/salmonella_main.html. 547 out of 869 interactions were present in the reference human interactome. Remaining 322 interactions were novel, predicted interactions.

To check if this reconstructed network is specific to *Salmonella* infection, we have searched for the known targets of *Salmonella* effectors in the network. In this step, we have used the interactome that has been curated and compiled from published studies where 40 effectors interact with 50 human proteins through 62 interactions (Schleker et al., 2012). We found that 13 proteins out of 50 were present in the reconstructed network, which are known to be the targets of *Salmonella* effectors. The enrichment of *Salmonella* effector targets in the reconstructed network is statistically significant when compared to the overall human interactome (*p* = 8.206×10^−8^, by hypergeometric test) which implies the specificity of the reconstructed network to *Salmonella* infection. In Table 1, targets of *Salmonella* effectors present in the reconstructed network are listed with their functions and whether they are phoshoproteomic hits or found by our approach as intermediates. Additionally, we have checked the Gene Ontology and KEGG pathway enrichments for the overall reconstructed network. Regulation of transcription (*p* = 2.0×10^−3^), apoptosis (*p* = 3.9×10^−10^), intracellular transport (*p* = 1.0×10^−8^), cell cycle (*p* = 2.1×10^−10^), cytoskeleton organization (*p* = 2.3×10^−17^) are some of the processes enriched in the reconstructed network. More specifically, SNARE interaction in vesicular transport (*p* = 1.0×10^−6^), mTOR signaling (*p* = 1.5×10^−4^), and MAPK signaling (*p* = 9.9×10^−6^) pathways are among the enriched pathways. In several studies, *Salmonella* infection was shown to down-regulate mTOR pathway to induce apoptosis (Lee et al., 2014). The *Salmonella* effector AvrA targets MAPK signaling, mTOR signaling, and NF-κB pathways to manipulate the processes in the host cell (Liu et al., 2010).

**Table 1:** **Targets of *Salmonella* effectors in the reconstructed network**.

**Host protein**	**Function**	**Pathogen effectors**	Type of the node in the network^*^
CDC42	Actin filament bundle assembly	SopB, SopE	intermediate
MTOR	Protein serine/threonine kinase activity	AvrA	intermediate
RHOA	Actin cytoskeleton organization	SifA, SseJ	intermediate
YWHAG	Negative regulation of protein serine/threonine kinase activity	SspH2	intermediate
TP53	Apoptotic activity	AvrA	intermediate
TLN1	Structural constituent of cytoskeleton	SseL	intermediate
KLC1	Microtubule motor activity	PipB2	pp-hit
FLNA	Actin cytoskeleton reorganization	SseI, SrfH, SspH2	pp-hit
CTNNB1	Cytoskeletal anchoring at plasma membrane	AvrA	pp-hit
VIM	Intermediate filament organization	SptP	pp-hit
IQGAP1	Ras GTPase activator activity	SseI, SrfH	pp-hit
JUP	Cytoskeletal anchoring at plasma membrane	SseF	pp-hit
KRT18	Intermediate filament cytoskeleton organization	SipC or SspC	pp-hit
MAPK1	Activation of MAPK activity	AvrA, SpvC	pp-hit
OSBPL9, OSBPL10, OSBPL11	Lipid transport	SseL	pp-hit (except OSBPL11)
STX1A, STX3, STX4, STX5, STX7, STX12, STXBP5	Intracellular protein transport		intermediate (except STX7, STX12)

* Intermediate, Steiner node; pp-hit, Phosphoproteomic hit.

Next, the reconstructed network is divided into 20 clusters using the RNSC algorithm, which searches highly connected node sets within a given graph. Each cluster was found to be enriched in few specific biological processes or pathways. Top three most significant processes are listed in Table 2. For example, the first cluster is enriched in the mTOR signaling pathway, cytoskeleton organization, and other processes. Also enrichments in the intracellular transport, apoptosis, RNA processing, and transcription is observed in different clusters. These clusters have also been analyzed based on the enrichment of cellular components, and number of clusters observed in three cellular compartments; nucleus, cytosol and cytoskeleton is reported in Figure S2.

**Table 2:** **Gene ontology (GO) biological process enrichments of each cluster located in the final network**.

**Cluster #**	**GO Term**	***p*-values**	**Percent**
1	Cytoskeleton organization	0.00180	1.65
	Regulation of cellular component size	0.00220	1.37
	Regulation of cytoskeleton organization	0.00270	1.1
2	Cytoskeleton organization	0.00906	1.5
	Regulation of phosphorylation	0.01138	1.5
3	Protein amino acid phosphorylation	0.01509	1.45
	Phosphorylation	0.02753	1.45
4	RNA processing	0.0013	1.94
	Intracellular signaling cascade	0.00152	2.77
	Cell cycle process	0.00155	1.94
5	Not any significant GO enrichment		
6	Intracellular transport	0.00233	1.65
	Membrane organization	0.00842	1.18
	Negative regulation of macromolecule metabolic process	0.0186	1.41
7	Response to organic cyclic substance	0.001745	1.03
	Negative regulation of macromolecule metabolic process	0.002774	1.81
	Regulation of cell cycle	0.00395	1.29
8	Positive regulation of specific transcription from RNA polymerase II promoter	0.00381	1.05
	Cell death	0.005171	2.11
	Death	0.005325	2.11
9	RNA processing	0.002175	1.72
	Transmembrane receptor protein tyrosine kinase signaling pathway	0.0022984	1.23
	Cytoskeleton organization	0.004371	1.47
10	Regulation of small GTPase mediated signal transduction	3.3120E-9	1.13
	Small GTPase mediated signal transduction	3.52554E-7	1.01
	Intracellular signaling cascade	5.2661E-4	1.13
11	Regulation of cellular protein metabolic process	0.00220	1.57
	Response to organic substance	0.002536	1.83
	Response to hormone stimulus	0.005706	1.31
12	Actin filament-based process	0.008572	1.1
	Cell cycle phase	0.035767	1.1
	Cytoskeleton organization	0.04074	1.1
13	Negative regulation of programmed cell Death	0.00105	1.73
	Negative regulation of cell death	0.00106	1.73
	Negative regulation of cell proliferation	0.007898	1.45
14	M phase	0.003363	1.12
	Cell cycle phase	0.007577	1.12
	Chromosome organization	0.013039	1.12
15	Protein import	0.001547	1.21
	Protein localization in organelle	0.0021107	1.21
	Intracellular transport	0.005237	1.82
16	Macromolecular complex assembly	0.012584	1.04
	Macromolecular complex subunit organization	0.0163	1.04
	Regulation of apoptosis	0.02648	1.04
17	Tube development	0.005909	1.38
	Tube morphogenesis	0.01944	1.04
	Cellular component morphogenesis	0.0287436	1.38
18	Regulation of gene-specific transcription	0.00186	1.29
	Positive regulation of macromolecule metabolic process	0.00397	2.26
	Positive regulation of macromolecule biosynthetic process	0.00617	1.94
19	Positive regulation of molecular function	0.016011	1.69
	Positive regulation of nucleobase, nucleoside, nucleotide, and nucleic acid metabolic process	0.019733	1.69
	Positive regulation of nitrogen compound metabolic process	0.021893988499693085	1.69
20	Not any significant GO enrichment		

The percent column has been calculated by dividing the number of involved proteins in that GO term to the total number of proteins in that cluster and converted into percentage.

With the help of network analysis techniques, we were able to rank the nodes present in the reconstructed network. The most central nodes (based on different measures) are listed in Table 3 where 10 proteins that have known functions in apoptosis are observed. 14-3-3ζ (YWHAZ) and p53 (TP53) are the most frequently observed proteins on the simple paths passing through the hits from 2 min to 20 min. MAPK1, CDC42, MAP3K3, and 14-3-3δ (YWHAG) behave like signal transducers where the number of incoming edges are very low compared to other nodes; while MAPK1 also behaves like a signal receiver when the number of edges of each node were compared. These proteins are critical for the structure of the network; in other words, these proteins have the potential to reveal clinically important targets.

**Table 3:** **Top ranking proteins in the reconstructed network**.

**Name**	**Function**	**Subcellular location**	**Path frequency**	**In degree**	**Out degree**	**Betweenness**
ACTG1	Structural constituent of cytoskeleton	Actin cytoskeleton	7.3	2	5	0.0017
AIFM1	Apoptotic process	Mitochondrion	6.8	1	1	0.0017
APBB2	Beta-amyloid binding	Cytoplasm	0.0	1	24	0.0001
ATXN1	DNA binding	Cytoplasm, nucleus	14.8	8	5	0.0021
CDC42	Actin cytoskeleton organization	Cytoskeleton	4.2	1	13	0.0002
CFL1	Actin cytoskeleton organization	Cytoskeleton	24.3	8	1	0.001
CIC	DNA binding	Nucleus	9.5	5	1	0.0008
CRTC3	cAMP response element binding protein binding	Cytoplasm, nucleus	12.7	1	0	0.0
CTNNB1	Alpha-catenin binding	Cytoskeleton	0.0	2	9	0.0008
EIF2AK2	Protein serine/threonine kinase activity	Cytoplasm, nucleus	1.6	6	2	0.001
EIF3G	Translation initiation factor activity	Cytoplasm, nucleus	6.8	2	2	0.002
EIF4G1	Translation factor activity, nucleic acid binding	Cytosol, membrane	37.6	8	6	0.004
HSPB1	Cellular component movement	Cytoskeleton	22.0	3	2	0.0012
MAP3K3	Activation of MAPKK activity	Cytosol	0.0	0	9	0.0
MAPK1	MAP kinase activity	Cytoskeleton	11.1	12	1	0.0015
MPP6	Maturation of 5.8S rRNA	Membrane	9.8	2	2	0.002
MPZL1	Cell-cell signaling	Membrane	24.9	3	1	0.0005
RELA	Sequence-specific DNA binding transcription factor activity	Nucleus	0.0	3	11	0.0016
SRC	Protein tyrosine kinase activity	Membrane, cytoskeleton	2.1	3	13	0.0006
TOP2A	Chromatin binding	Cytoplasm, nucleus	0.0	6	1	0.0006
TP53	Tumor suppressor; induces growth arrest or apoptosis	Cytoplasm, nucleus	32.8	15	14	0.011
UBE2I	SUMO ligase activity	Cytoplasm, nucleus	7.3	4	8	0.0016
YWHAG	Protein kinase binding	Cytoplasmic vesicle membrane	0.0	0	13	0.0
YWHAZ	Protein kinase binding	Cytoplasmic vesicle membrane	65.1	34	11	0.0093
ZC3HAV1	Cellular response to exogenous dsRNA	Cytoplasm, nucleus	24.9	1	0	0.0

### CDC42 is a clinically important target in *salmonella* infection

**Figure 3:**
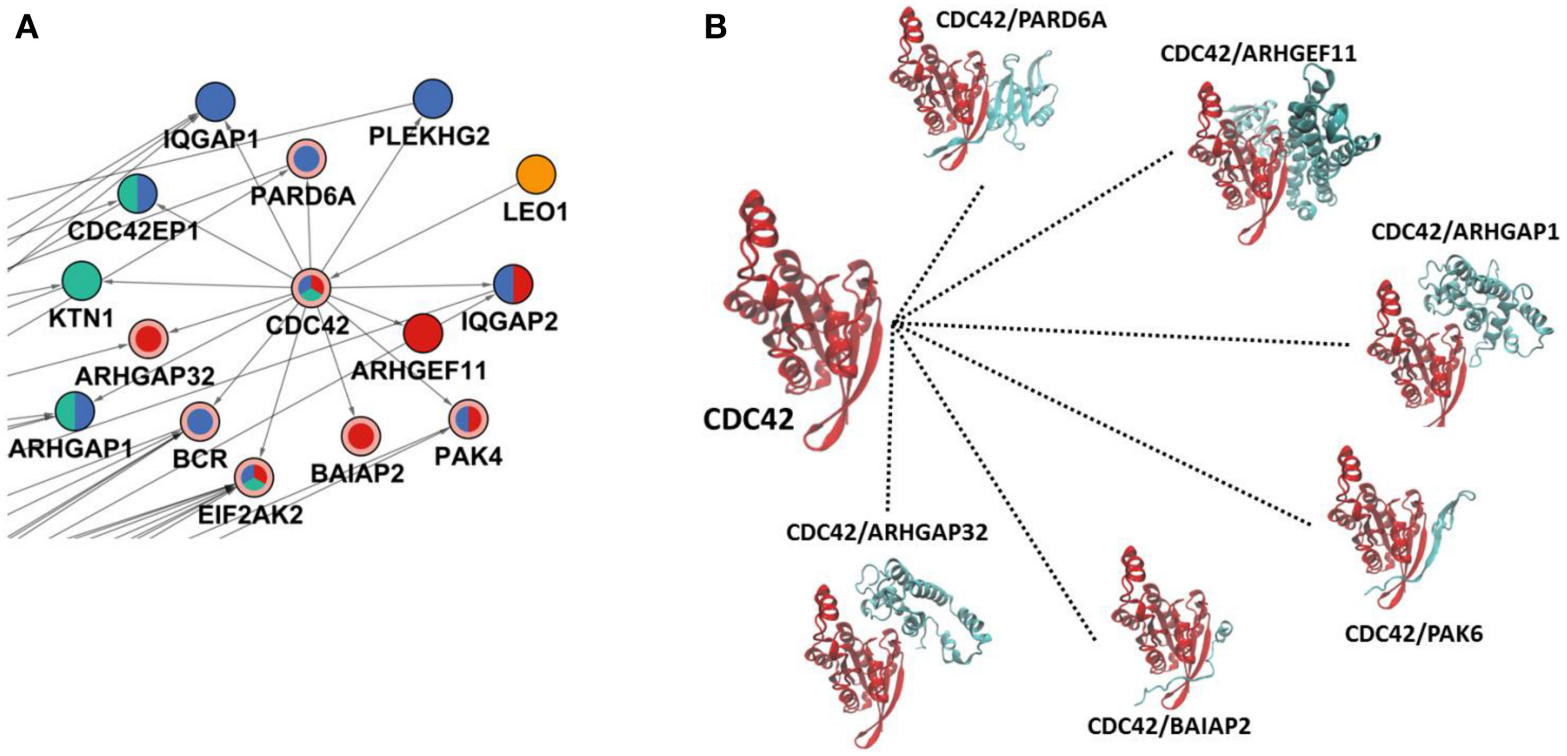
**CDC42 and its interactions in the reconstructed network**. **(A)** The region where CDC42 and its first neighbors are located in the reconstructed network. The coloring scheme is the same as in Figure 2 where CDC42, EIF2AK2, BCR, BAIAP2, ARHGAP32, PARD6A, and PAK4 are Steiner nodes and others are phosphoproteomic hits. **(B)** Structural details of CDC42 interactions where CDC42 uses the same binding site completely or partially to interact with its partners. Here, PAK6 is a structural homolog of PAK4; therefore to show similar binding of PAK4, it is represented with PAK6.

Two effectors of *Salmonella*, SopE, and SopB, stimulate CDC42 (cell division control protein 42) protein which induces rearrangements in the cytoskeleton. CDC42 were not present in the set of phosphoproteomic hits; however, it was located in a central part of the reconstructed network, which is revealed by the PCSF approach. Although CDC42 has a phosphorylation site at tyrosine 64 (Tu et al., 2003) which is not present in the initial phosphoproteomic data, our approach correctly locates CDC42 in the final reconstructed network. Based on the total degrees, CDC42 is among the top 10 ranking proteins. In the reconstructed network, CDC42 has only one incoming edge, but has 13 outgoing edges which is consistent with the infection mechanism of *Salmonella* where stimulation of CDC42 leads to activation of many downstream signaling components including p21-activated kinases (PAKs) and PBD domain containing proteins (Galan and Zhou, 2000). PAK4 and PBD domain containing protein CDC42EP1 are among the downstream partners in the reconstructed network. Also, when we zoomed into the first degree neighbors of CDC42, their downstream components in our network can be observed (see Figure 3A). Some of these partners are active at 5 min, or at 10 min, or at other time points. The CDC42 shows a hub-like character in the reconstructed network. Hub proteins cannot interact with all their partners at the same time. They either adapt multiple binding sites or use a single binding site repeatedly. This property of hubs has been well-established for TP53 protein where four binding sites are repeatedly used to interact with different partners (Tuncbag et al., 2009). We have checked this property in CDC42 to understand its interactions by searching for the available structural data in Protein Databank (PDB) (Berman et al., 2000) and Interactome3D (Mosca et al., 2013). We have found six interactions out of 13 in atomic detail (see Figure 3B). Structural data provides information about at which region two proteins are interacting. Analysis of the binding site for each protein pair has shown that CDC42 is using the same binding region completely or partially to interact with its partners and this property is a characteristic of hub proteins. Also, the downstream partners of CDC42 are active at different time points as illustrated in Figure 3A, which also shows the mutually exclusive character of the interactions. For example, PAK4 is in the reconstructed network showing its effect after infection at time points 10 and 20 min. PARD6A and ARHGAP32 effect at 10 and 20 min, respectively. The partner proteins are effective at different time points and CDC42 can bind to these proteins in a mutually exclusive manner. IQGAP1 is another downstream component, which promotes *Salmonella* invasion by binding to CDC42 and knockdown of IQGAP1 was shown to be reducing the invasion (Brown et al., 2007).

### The reconstructed signaling network revealed many other potential clinical targets

Besides CDC42, some other targets of *Salmonella* effectors were located correctly in the reconstructed network although they were not present in the initial phosphoproteomic data; such as 14-3-3δ, RHOA, TP53, TLN1 (Schleker et al., 2012). Also pathogen targets such as β-catenin, MAPK1, IQGAP1 are observed in the reconstructed network as phosphoproteomic hits (Schleker et al., 2012).

**Figure 4:**
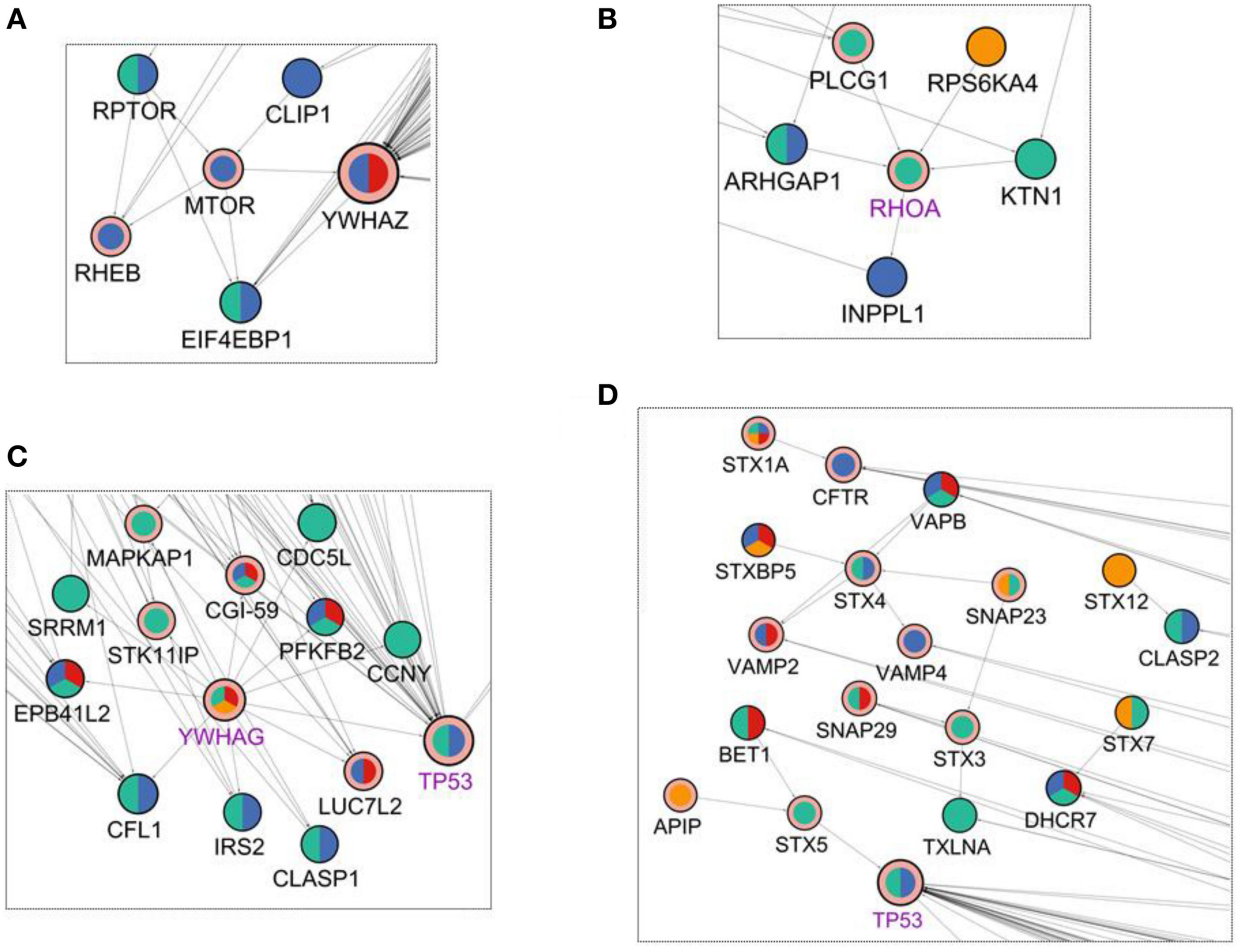
**Visualization of the first degree neighbors of (A) mTOR, (B) RHOA, (C) YWHAG, and (D) Syntaxins in the reconstructed network in Figure 2**. The coloring scheme is the same as in Figure 2.

In addition to these targets, mTOR pathway was found to be enriched in the reconstructed network. mTOR signaling was known to be altered after *Salmonella* infection and mTOR is a phosphoproteomic hit having significant effect at 10 min in our data. When we have investigated the neighbors of the mTOR protein (Figure 4A) RHEB, a direct regulator of mTOR (Long et al., 2005), is observed as an interactor of mTOR in the reconstructed network. Also, RPTOR binding to mTOR and EIF4EBP1 was recovered in our network. The mTOR - RHEB complex induces phosphorylation of EIF4EBP1 (Long et al., 2005). Even though RHEB is an important player in the activation of EIF4EBP1, it was not observed within the initial phosphoproteomic hits. The proposed two-step modeling approach was able to locate RHEB in the final network, completing the missing interactions of the signaling pathway. According to our network, signaling in the RPTOR-mTOR-Rheb-EIF4EBP1 axis starts at 5 min and continues until 10 min.

RHOA, is a GTPase that functions in the actin cytoskeleton organization (Hall, 1998). It was a Steiner node in final network as a target of *Salmonella* effectors. It has 5 binding partners in the reconstructed network of where four of them are upstream interactors and only one is a downstream interactor (Figure 4B). Among the upstream interactors, RPS6KA4 is effective at 2 min and the remaining ones are active at 5 min. The downstream interactor INPPL1 is effective at 10 min. So, the final reconstructed network suggests that RHOA receives signals from proteins active at min 2 and min 5 and transmits these signals until min 10.

The 14-3-3δ protein (YWHAG) shows a pattern similar to CDC42 where the incoming interactions are not present, but there are many outgoing interactions from 14-3-3δ which implies that 14-3-3δ is a signal mediator for the downstream components of the network. In Figure 4C, first neighbors of 14-3-3δ are illustrated in the network. 14-3-3δ is a Steiner node, a known target of *Salmonella* effectors, and it is effective at min 2, 5, and 20. Its 13 outgoing edges suggest a function like a signal transducer, sending signals to many downstream proteins at different time points.

Also, seven proteins from the Syntaxin family were present in the reconstructed network and only three of them was a phosphoproteomic hit, others were Steiner nodes found by our approach. Syntaxins function in vesicle trafficking which is an important process in *Salmonella* replication and transport. *Salmonella* effectors hijack syntaxins by binding them (Ramos-Morales, 2012).

Finally, oxysterol-binding proteins (OSBPs) are also present in the reconstructed network, which are known to be enhancing replication of *Salmonella* in the host cell by interacting with the *Salmonella* effector SseL (Auweter et al., 2012). This interaction can lead to the exploitation of OSBP dependent pathways altered during the *Salmonella* infection.

## Discussion

Improvements in the high-throughput technologies revolutionized the systems biology era. Instead of comparing lists of genes or proteins, finding the interactions, regulations, and mechanisms within a set of significantly altered proteins or genes have gained importance. In addition, integration of multiple “omic” hits in a biologically meaningful way is now crucial to better understand the functional pathways and cellular mechanisms that are active during a disease or perturbation. For this purpose, several network modeling approaches have been developed, which successfully reveals clinically valuable targets and important pathways, especially in several cancer types. Another dimension of omic data is its temporality, which makes the network modeling process more challenging, as instead of simply connecting omic hits, the time related constraints have to be considered during network reconstruction.

In this work, we have provided a proof-of-concept application of an integrative approach which benefits from two different network reconstruction methods, namely PCSF and ILP based edge inference methods. Although both methods infer networks from experimental omic hits, they perform better in different parts of the modeling. The former reveals the hidden components of the signaling, but cannot handle time as a dimension. The latter can integrate temporal information to reconstruct directed edges, but cannot add missing signaling components to complete the lacking parts of the signaling. These complementary aspects of the methods inspired us to combine both approaches to model the signaling network of human cells after *Salmonella* infection based on the temporal phosphoproteomic data. We have selected *Salmonella* infection, because the signaling changes in the host cells are still unknown despite the efforts to understand the communication details between the pathogen and the host. In addition, available approaches have not been yet applied to model signaling changes in the host organism during *Salmonella* infection. In the first stage, we have integrated the temporal phosphoprotemic data of *Salmonella*-infected human cells with confidence weighted protein–protein interactions to reconstruct the signaling pathway for each time point with the PCSF approach. Then, all the components were labeled with the corresponding time points based on their presence in the reconstructed networks, and used as the input for the ILP-based edge inference step, in which directions are assigned to the interactions based on the temporal data. The final network with 658 proteins and 869 interactions provided a rich source to analyze the signaling alterations and clinical target identification. Our approach allowed us to identify host pathways functioning during the *Salmonella* infection and to rank the proteins according to their importance for the infection based on their centrality in the network. The resulting network conserves the information about temporality, direction of interactions, while revealing the hidden entities in the signaling. Several pathways such as SNARE binding, mTOR signaling, immune response, cytoskeleton organization, and apoptosis, were found to be effected, many of which were previously found to be altered in the host cell after *Salmonella* infection. Additionally, we have shown that the reconstructed network is enriched in the protein targets of the *Salmonella* effectors. Clustering of the resulting network showed that the multiple biological processes are enriched in each cluster. The final network also involves enrichments in the cytoskeletal organization and the regulation of cellular component size. These findings are in parallel with the known infection mechanism of the *Salmonella* where the injected effector proteins trigger the epithelial cell membrane by rearranging the cytoskeleton of the host cell that results in invasion of the bacterium into the host cell.

Another benefit of the proposed two-step approach (Figure 1) was that hidden components of signaling can be revealed with network reconstruction. In this specific demonstration, several known targets of *Salmonella* effectors have been accurately included in the reconstructed network such as CDC42, RHOA, 14-3-3δ, Syntaxins although they were not present in the initial phosphoproteomic data. These hidden signaling components can be potential therapeutic targets. Among them CDC42 is a target of the effector protein SopB and their interaction helps in the adaptation of *Salmonella* to the intracellular condition of the host. CDC42 is responsible of downstream signaling and behaves as a signal transmitter. From a medical point of view, targeting CDC42 is a good approach both for blocking the adaptation of *Salmonella* in the host cell and abnormal downstream signaling during infection (Figure 3). RHOA functions in cytoskeleton organization and also it a target of *Salmonella* effectors. The effector SifA activates RHOA during infection. Activated RHOA promotes opening tubes in the membrane (Srikanth et al., 2010). Therefore, RHOA can be considered as a therapeutic target and controlling its activation can be a good approach in *Salmonella* treatment (Figure 4B). Also, besides revealing the hidden components, the reconstructed edge directions nicely showed how the signals are transmitted temporally from one layer to another. For example, some host proteins behave like a signal receiver such as MAPK1 and some others behave like a signal transmitter such as, 14-3-3δ (Figure 4C).

Understanding these communications and signaling details in the host is crucial to improve the available treatment strategies for *Salmonella* infection in the near future, especially as the new antibiotic-resistance species are on the rise. We believe that the integrated approaches, such as the one presented here, have a high potential for understanding the key molecular mechanisms in bacteria's susceptibility or resistance to the available antibiotics and for the identification of new clinical targets in infectious diseases, especially in the *Salmonella* infection.

## Conflict of interest statement

The authors declare that the research was conducted in the absence of any commercial or financial relationships that could be construed as a potential conflict of interest.

## References

[B1] Aijo T. Granberg K. Lahdesmaki H. (2013). Sorad: a systems biology approach to predict and modulate dynamic signaling pathway response from phosphoproteome time-course measurements. Bioinformatics 29, 1283–1291. 10.1093/bioinformatics/btt130 23505293

[B2] Auweter S. D. Yu H. B. Arena E. T. Guttman J. A. Finlay B. B. (2012). Oxysterol-binding protein (OSBP) enhances replication of intracellular Salmonella and binds the Salmonella SPI-2 effector SseL via its N-terminus. Microbes Infect. 14, 148–154. 10.1016/j.micinf.2011.09.003 21988961

[B3] Bailly-Bechet M. Borgs C. Braunstein A. Chayes J. Dagkessamanskaia A. Francois J. M.. (2010). Finding undetected protein associations in cell signaling by belief propagation. Proc. Natl. Acad. Sci. U.S.A. 108, 882–887. 10.1073/pnas.1004751108 21187432 PMC3021011

[B4] Bender C. Henjes F. Frohlich H. Wiemann S. Korf U. Beissbarth T. (2010). Dynamic deterministic effects propagation networks: learning signalling pathways from longitudinal protein array data. Bioinformatics 26, i596–i602. 10.1093/bioinformatics/btq385 20823327 PMC2935402

[B5] Berman H. M. Westbrook J. Feng Z. Gilliland G. Bhat T. N. Weissig H.. (2000). The Protein Data Bank. Nucleic Acids Res. 28, 235–242. 10.1093/nar/28.1.235 10592235 PMC102472

[B6] Brohee S. Faust K. Lima-Mendez G. Sand O. Janky R. Vanderstocken G.. (2008). NeAT: a toolbox for the analysis of biological networks, clusters, classes and pathways. Nucleic Acids Res. 36, W444–W451. 10.1093/nar/gkn336 18524799 PMC2447721

[B7] Brown M. D. Bry L. Li Z. Sacks D. B. (2007). IQGAP1 regulates Salmonella invasion through interactions with actin, Rac1, and Cdc42. J. Biol. Chem. 282, 30265–30272. 10.1074/jbc.M702537200 17693642

[B8] Dandekar T. Astrid F. Jasmin P. Hensel M. (2012). *Salmonella enterica*: a surprisingly well-adapted intracellular lifestyle. Front. Microbiol. 3:164. 10.3389/fmicb.2012.00164 22563326 PMC3342586

[B9] De Jong H. (2002). Modeling and simulation of genetic regulatory systems: a literature review. J. Comput. Biol. 9, 67–103. 10.1089/10665270252833208 11911796

[B10] Dhal P. K. Barman R. K. Saha S. Das S. (2014). Dynamic modularity of host protein interaction networks in Salmonella Typhi infection. PLoS ONE 9:e104911. 10.1371/journal.pone.0104911 25144185 PMC4140748

[B11] Dittrich M. T. Klau G. W. Rosenwald A. Dandekar T. Muller T. (2008). Identifying functional modules in protein-protein interaction networks: an integrated exact approach. Bioinformatics 24, i223–i231. 10.1093/bioinformatics/btn161 18586718 PMC2718639

[B12] Eckmann L. Smith J. R. Housley M. P. Dwinell M. B. Kagnoff M. F. (2000). Analysis by high density cDNA arrays of altered gene expression in human intestinal epithelial cells in response to infection with the invasive enteric bacteria Salmonella. J. Biol. Chem. 275, 14084–14094. 10.1074/jbc.275.19.14084 10799483

[B13] Eren Ozsoy O. Aydin Son Y. Can T. (2015). Reconstruction of Signaling and Regulatory Networks using Time-series and Perturbation Experiments. Technical Report, Department of Computer Engineering, Middle East Technical University.

[B14] Eren Ozsoy O. Can T. (2013). A divide and conquer approach for construction of large-scale signaling networks from PPI and RNAi data using linear programming. IEEE/ACM Trans. Comput. Biol. Bioinform. 10, 869–883. 10.1109/TCBB.2013.80 24334382

[B15] Frohlich H. Sahin O. Arlt D. Bender C. Beissbarth T. (2009). Deterministic effects propagation networks for reconstructing protein signaling networks from multiple interventions. BMC Bioinformatics 10:322 10.1186/1471-2105-10-322 19814779 PMC2770070

[B16] Galan J. E. Zhou D. (2000). Striking a balance: modulation of the actin cytoskeleton by Salmonella. Proc. Natl. Acad. Sci. U.S.A. 97, 8754–8761. 10.1073/pnas.97.16.8754 10922031 PMC34008

[B17] Gosline S. J. Spencer S. J. Ursu O. Fraenkel E. (2012). SAMNet: a network-based approach to integrate multi-dimensional high throughput datasets. Integr. Biol. (Camb). 4, 1415–1427. 10.1039/c2ib20072d 23060147 PMC3501250

[B18] Hall A. (1998). Rho GTPases and the actin cytoskeleton. Science 279, 509–514. 10.1126/science.279.5350.509 9438836

[B19] Hashemikhabir S. Ayaz E. S. Kavurucu Y. Can T. Kahveci T. (2012). Large-scale signaling network reconstruction. IEEE/ACM Trans. Comput. Biol. Bioinform. 9, 1696–1708. 10.1109/TCBB.2012.128 23221085

[B20] Hecker M. Lambeck S. Toepfer S. Van Someren E. Guthke R. (2009). Gene regulatory network inference: data integration in dynamic models—a review. Biosystems 96, 86–103. 10.1016/j.biosystems.2008.12.004 19150482

[B20a] Huang D. W. Sherman B. T. Lempicki R. A. (2009). Systematic and integrative analysis of large gene lists using DAVID bioinformatics resources. Nat. Protoc. 4, 44–57. 10.1038/nprot.2008.211 19131956

[B21] Huang S. S. Clarke D. C. Gosline S. J. C. Labadorf A. Chouinard C. R. Gordon W.. (2012). Linking proteomic and transcriptional data through the interactome and epigenome reveals a map of oncogene-induced signaling. PLoS Comp Biol. 9:e1002887. 10.1371/journal.pcbi.1002887 23408876 PMC3567149

[B22] Huang S. S. Fraenkel E. (2009). Integrating proteomic, transcriptional, and interactome data reveals hidden components of signaling and regulatory networks. Sci.Signal. 2, ra40. 10.1126/scisignal.2000350 19638617 PMC2889494

[B23] Kiani N. A. Kaderali L. (2014). Dynamic probabilistic threshold networks to infer signaling pathways from time-course perturbation data. BMC Bioinformatics 15:250. 10.1186/1471-2105-15-250 25047753 PMC4133630

[B24] Kim Y. A. Wuchty S. Przytycka T. M. (2011). Identifying causal genes and dysregulated pathways in complex diseases. PLoS Comput. Biol. 7:e1001095. 10.1371/journal.pcbi.1001095 21390271 PMC3048384

[B25] Knapp B. Kaderali L. (2013). Reconstruction of cellular signal transduction networks using perturbation assays and linear programming. PLoS ONE 8:e69220. 10.1371/journal.pone.0069220 23935958 PMC3728289

[B26] Lawhon S. D. Khare S. Rossetti C. A. Everts R. E. Galindo C. L. Luciano S. A. (2011). Role of SPI-1 secreted effectors in acute bovine response to *Salmonella enterica* Serovar Typhimurium: a systems biology analysis approach. PLoS ONE 6:e26869 10.1371/journal.pone.0026869 22096503 PMC3214023

[B27] Lee C. H. Lin S. T. Liu J. J. Chang W. W. Hsieh J. L. Wang W. K. (2014). Salmonella induce autophagy in melanoma by the downregulation of AKT/mTOR pathway. Gene Ther. 21, 309–316. 10.1038/gt.2013.86 24451116

[B28] Linde J. Schulze S. Henkel S. Guthke R. (2015). Data- and knowledge-based modeling of gene regulatory networks: an update. EXCLI J. 346–378. 10.17179/excli2015-168 PMC4817425 27047314

[B29] Liu X. Lu R. Xia Y. Wu S. Sun J. (2010). Eukaryotic signaling pathways targeted by Salmonella effector protein AvrA in intestinal infection *in vivo*. BMC Microbiol. 10:326. 10.1186/1471-2180-10-326 21182782 PMC3027599

[B30] Long X. Lin Y. Ortiz-Vega S. Yonezawa K. Avruch J. (2005). Rheb binds and regulates the mTOR kinase. Curr. Biol. 15, 702–713. 10.1016/j.cub.2005.02.053 15854902

[B31] Markowetz F. Kostka D. Troyanskaya O. G. Spang R. (2007). Nested effects models for high-dimensional phenotyping screens. Bioinformatics 23, i305–i312. 10.1093/bioinformatics/btm178 17646311

[B32] Matos M. R. Knapp B. Kaderali L. (2015). lpNet: a linear programming approach to reconstruct signal transduction networks. Bioinformatics. [Epub ahead of print]. 10.1093/bioinformatics/btv327 26026168

[B33] Melas I. N. Samaga R. Alexopoulos L. G. Klamt S. (2013). Detecting and removing inconsistencies between experimental data and signaling network topologies using integer linear programming on interaction graphs. PLoS Comput. Biol. 9:e1003204 10.1371/journal.pcbi.1003204 24039561 PMC3764019

[B34] Mosca R. Ceol A. Aloy P. (2013). Interactome3D: adding structural details to protein networks. Nat. Methods 10, 47–53. 10.1038/nmeth.2289 23399932

[B35] Raghunathan A. Reed J. Shin S. Palsson B. Daefler S. (2009). Constraint-based analysis of metabolic capacity of *Salmonella typhimurium* during host-pathogen interaction. BMC Syst. Biol. 3:38. 10.1186/1752-0509-3-38 19356237 PMC2678070

[B36] Ramos-Morales F. (2012). Impact of *Salmonella enterica* type III secretion system effectors on the eukaryotic host cell. ISRN Cell Biol. 2012, 1–36. 10.5402/2012/78793424858684

[B37] Rogers L. D. Brown N. F. Fang Y. Pelech S. Foster L. J. (2011). Phosphoproteomic analysis of Salmonella-infected cells identifies key kinase regulators and SopB-dependent host phosphorylation events. Sci. Signal. 4, rs9. 10.1126/scisignal.2001668 21934108

[B38] Schleker S. Sun J. Raghavan B. Srnec M. Müller N. Koepfinger M.. (2012). The current Salmonella-host interactome. Proteomics. Clin. Appl. 6, 117–133. 10.1002/prca.201100083 22213674 PMC3312997

[B39] Schult D. A. Swart P. (2008). Exploring network structure, dynamics, and function using NetworkX, in Proceedings of the 7th Python in Science Conferences (SciPy 2008), eds Varoquaux G. Vaught T. Millman J. (Pasadena, CA), 11–16.

[B40] Srikanth C. Wall D. M. Maldonado-Contreras A. Shi H. N. Zhou D. Demma Z.. (2010). Salmonella pathogenesis and processing of secreted effectors by caspase-3. Science 330, 390–393. 10.1126/science.1194598 20947770 PMC4085780

[B41] Steele-Mortimer O. (2011). Exploitation of the ubiquitin system by invading bacteria. Traffic 12, 162–169. 10.1111/j.1600-0854.2010.01137.x 20977569 PMC3038682

[B42] Stolovitzky G. Monroe D. Califano A. (2007). Dialogue on Reverse−Engineering Assessment and Methods. Ann. N. Y. Acad. Sci. 1115, 1–22. 10.1196/annals.1407.021 17925349

[B43] Taylor R. C. Singhal M. Weller J. Khoshnevis S. Shi L. Mcdermott J. (2009). A network inference workflow applied to virulence-related processes in Salmonella typhimurium. Ann. N. Y. Acad. Sci. 1158, 143–158. 10.1111/j.1749-6632.2008.03762.x 19348639

[B44] Tu S. Wu W. J. Wang J. Cerione R. A. (2003). Epidermal growth factor-dependent regulation of Cdc42 is mediated by the Src tyrosine kinase. J. Biol. Chem. 278, 49293–49300. 10.1074/jbc.M307021200 14506284

[B45] Tuncbag N. Braunstein A. Pagnani A. Huang S. S. Chayes J. Borgs C.. (2013). Simultaneous reconstruction of multiple signaling pathways via the prize-collecting steiner forest problem. J. Comput. Biol. 20, 124–136. 10.1089/cmb.2012.0092 23383998 PMC3576906

[B46] Tuncbag N. Kar G. Gursoy A. Keskin O. Nussinov R. (2009). Towards inferring time dimensionality in protein-protein interaction networks by integrating structures: the p53 example. Mol. Biosyst. 5, 1770–1778. 10.1039/b905661k 19585003 PMC2898629

[B47] Turner B. Razick S. Turinsky A. L. Vlasblom J. Crowdy E. K. Cho E.. (2010). iRefWeb: interactive analysis of consolidated protein interaction data and their supporting evidence. Database 2010:baq023. 10.1093/database/baq023 20940177 PMC2963317

[B48] Yeger-Lotem E. Riva L. Su L. J. Gitler A. D. Cashikar A. G. King O. D.. (2009). Bridging high-throughput genetic and transcriptional data reveals cellular responses to alpha-synuclein toxicity. Nat. Genet. 41, 316–323. 10.1038/ng.337 19234470 PMC2733244

[B49] Yoon H. McDermott J. E. Porwollik S. McClelland M. Heffron F. (2009). Coordinated regulation of virulence during systemic infection of *Salmonella enterica* serovar Typhimurium. PLoS Pathog. 5:e1000306. 10.1371/journal.ppat.1000306 19229334 PMC2639726

