# Supplementary material for: Reconstruction of the temporal signaling network in Salmonella-infected human cells
Source: Front Microbiol. 2015 Jul 20;6:730. doi: 10.3389/fmicb.2015.00730 (PMC4507143; doi:10.3389/fmicb.2015.00730)
Supplement: Supplementary file 1 [file DataSheet1.DOCX]

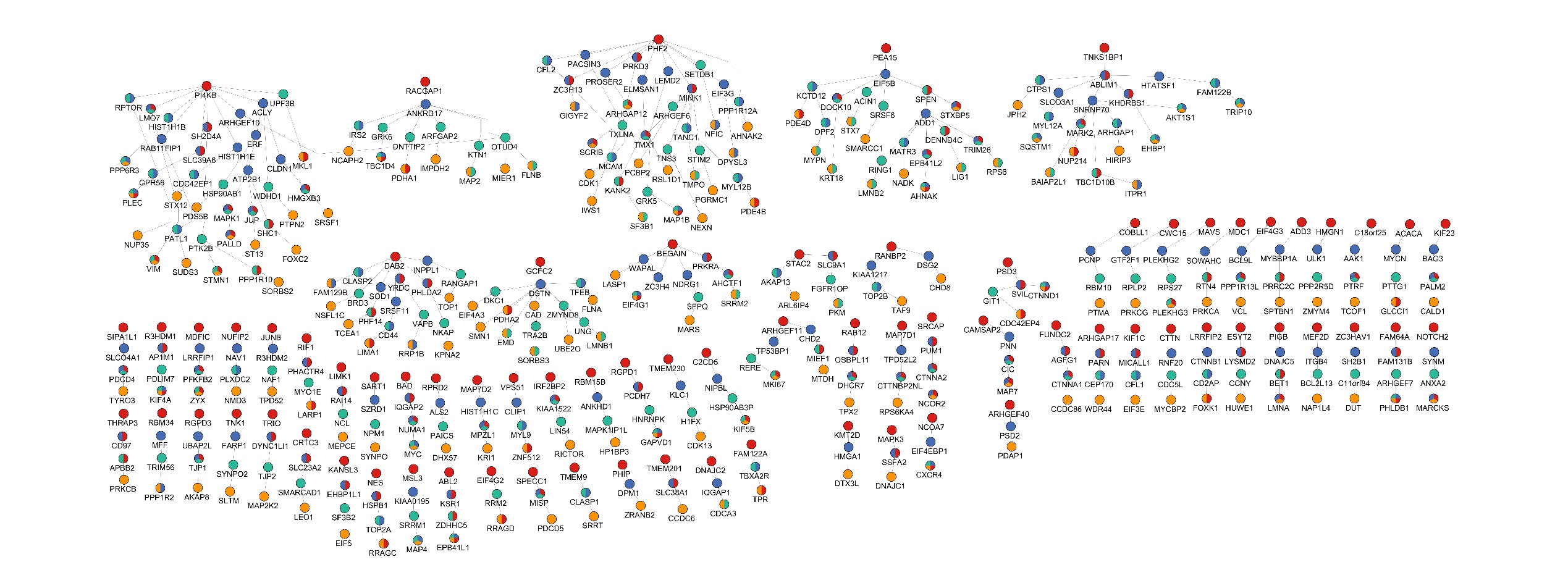


**Figure S1.** Visualization of directed network containing only phosphoproteomic hits. Only ILP-based edge inference approach has been applied to construct this network. Time points when any node is found to be critically changed are shown with different colors on the nodes; yellow indicates change in the node at 2 minutes, green indicates change in the node at 5 minutes, blue indicates change in the node at 10 minutes, and red indicates change in the node at 20 minutes. If a node is seen at more than one time points, then its coloring is done by a combination of colors of the corresponding time points.


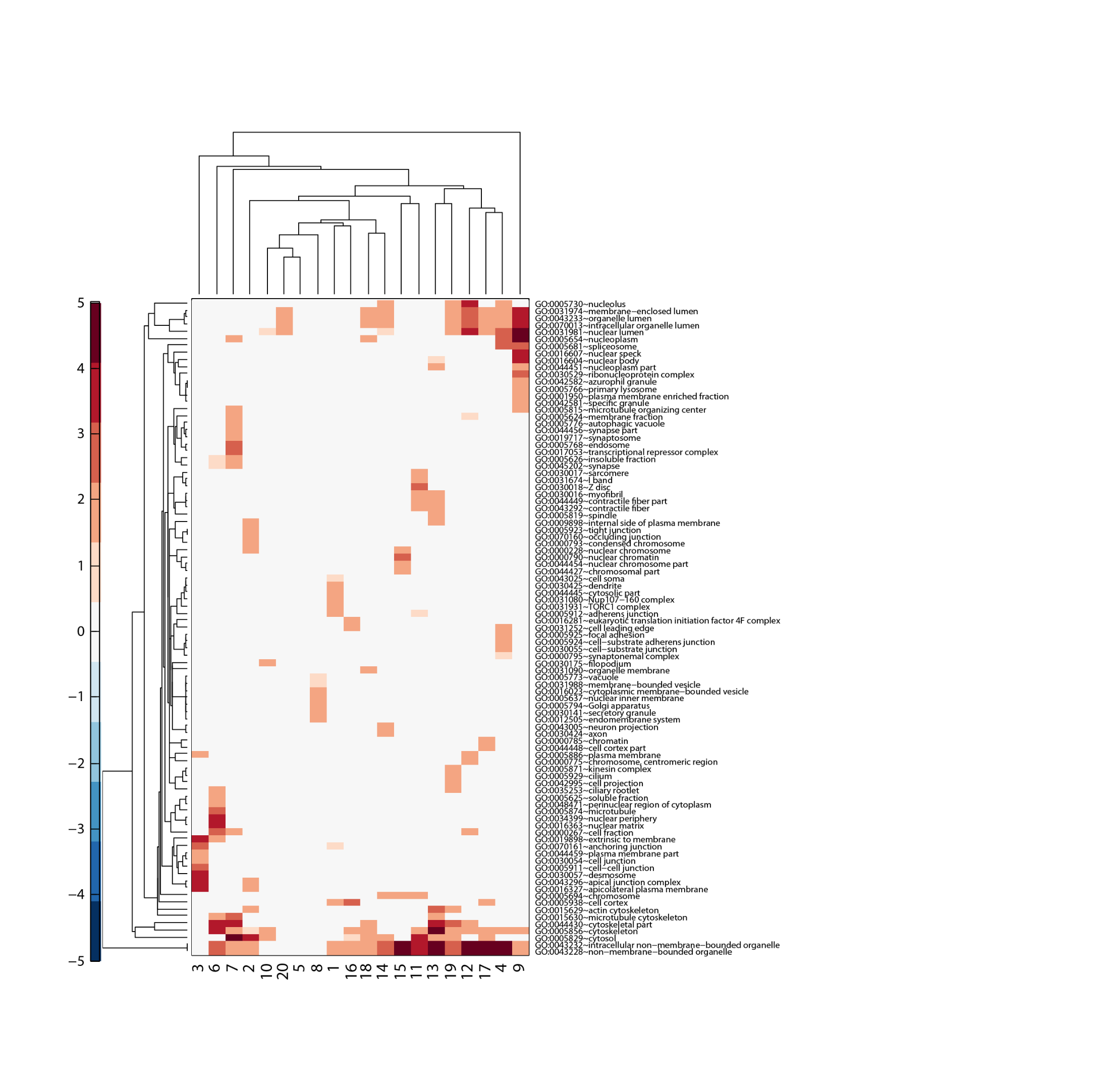


**Figure S2.** Cellular localization enrichment of each cluster in the reconstructed network (Figure 2 in the main text). Each column represents a clusters and each row represents a cellular localization. Entries are –log10 of the *p*-value of each cellular localization.

**Table S1.** Representing overlapping proteins in different time points and different locations. 2, 5, 10, and 20 are time points in minutes. M; membrane, C: cytoplasm, N: nucleus

|  | | 2 | | | 5 | | | 10 | | | 20 | | |
| --- | --- | --- | --- | --- | --- | --- | --- | --- | --- | --- | --- | --- | --- |
|  |  | M | C | N | M | C | N | M | C | N | M | C | N |
| 2 | M | 9 | 0 | 0 | 1 | 0 | 0 | 0 | 0 | 0 | 1 | 0 | 0 |
|  | C | 0 | 10 | 0 | 0 | 1 | 0 | 0 | 0 | 0 | 0 | 2 | 0 |
|  | N | 0 | 0 | 23 | 0 | 0 | 2 | 0 | 0 | 2 | 0 | 0 | 3 |
| 5 | M | 1 | 0 | 0 | 15 | 0 | 5 | 4 | 0 | 2 | 0 | 0 | 2 |
|  | C | 0 | 1 | 0 | 0 | 17 | 0 | 0 | 2 | 0 | 0 | 4 | 0 |
|  | N | 0 | 0 | 2 | 5 | 0 | 38 | 3 | 2 | 10 | 1 | 0 | 4 |
| 10 | M | 0 | 0 | 0 | 4 | 0 | 3 | 17 | 1 | 3 | 1 | 1 | 5 |
|  | C | 0 | 0 | 0 | 0 | 2 | 2 | 1 | 24 | 3 | 2 | 4 | 0 |
|  | N | 0 | 0 | 2 | 2 | 0 | 10 | 3 | 3 | 34 | 2 | 2 | 6 |
| 20 | M | 1 | 0 | 0 | 0 | 0 | 1 | 1 | 2 | 2 | 9 | 2 | 1 |
|  | C | 0 | 2 | 0 | 0 | 4 | 0 | 1 | 4 | 2 | 2 | 30 | 2 |
|  | N | 0 | 0 | 3 | 2 | 0 | 4 | 5 | 0 | 6 | 1 | 2 | 42 |
